# Artificial Intelligence Research for Complex Biological Systems: Integrating Data, Models, and Biological Knowledge

**DOI:** 10.3390/biology15141137

**Published:** 2026-07-13

**Authors:** Yong Chen, Milana Frenkel-Morgenstern

**Affiliations:** 1 Department of Biological and Biomedical Sciences, Rowan University, Glassboro, NJ 08028, USA; 2 Scojen Institute of Synthetic Biology, Dina Recanati School of Medicine, Reichman University, Tel Aviv District, Herzliya 4610101, Israel; milana.morgenstern@runi.ac.il

## 1. Introduction

Biological systems are inherently complex, dynamic, heterogeneous, and organized across multiple spatial and temporal scales. From molecular interactions and gene regulatory networks to cellular populations, tissues, organisms, and ecosystems, biological processes arise through nonlinear interactions among large numbers of components. Recent advances in high-throughput sequencing, single-cell technologies, spatial omics, imaging and structural biology have generated unprecedented volumes of biological data. However, the scale, dimensionality, noise, and heterogeneity of these datasets often exceed the capabilities of conventional analytical approaches.

Artificial intelligence (AI) and machine learning (ML) have become increasingly important for extracting meaningful patterns from complex biological data. Deep neural networks, transformers, graph-based models, generative models, matrix factorization, neural ordinary differential equations and hybrid biology-informed frameworks provide new opportunities, enabling the characterization of biological organization, prediction of molecular and cellular behavior, identification of therapeutic targets and generation of experimentally testable hypotheses. Importantly, the most useful AI approaches in biology must go beyond achieving high predictive accuracy. They should also incorporate biological knowledge, provide interpretable outputs, generalize across experimental conditions, and support rigorous biological and experimental validation.

This Special Issue, titled “Artificial Intelligence Research for Complex Biological Systems”, was established to present recent methodological and applied advances at the intersection of AI, bioinformatics, computational systems biology, genomics and biomedical research. The contributions cover a broad spectrum of biological scales and data modalities, including single-cell perturbation data, chromatin interaction maps, genomic sequences, drug–disease networks, biological images, protein antigens and multi-omics information. Together, these studies illustrate both the transformative potential of AI and the methodological challenges that must be addressed to ensure reliable and biologically meaningful discovery.

## 2. AI Across Biological Scales

A comprehensive review by Hein et al. [1] provides a broad overview of AI and ML applications spanning genes, genomes, proteins and integrated biological systems. The authors discuss developments ranging from conventional neural networks to transformers, graph neural networks, large language models and generative AI. The review highlights applications in gene function prediction, variant interpretation, protein structure prediction, molecular interaction modeling, multi-omics integration, personalized medicine and drug discovery. It also identifies persistent challenges involving data quality, model interpretability, ethical considerations and computational requirements. This review provides a useful conceptual framework for understanding the more specialized methodological contributions included in the Special Issue.

Several contributions focus on genomic and single-cell data, where sparsity, high dimensionality and experimental noise create substantial computational challenges. Yi et al. developed LazyNet, an interpretable neural ordinary differential equation framework for analyzing sparse single-cell CRISPR activation and interference screens [2]. Rather than relying on highly parameterized foundation models, LazyNet uses a compact log-linear exponential residual structure to represent multiplicative perturbation effects. The work demonstrates that computational efficiency and biological interpretability can be achieved without necessarily relying on very large pretrained models, particularly in low-data or resource-constrained settings.

Gao et al. introduced HiCENT, a generative transformer model designed to enhance both bulk and single-cell Hi-C contact matrices [3]. Hi-C technologies measure three-dimensional chromatin interactions, but insufficient sequencing depth and experimental noise often generate sparse, low-resolution contact maps. HiCENT learns structural dependencies within chromatin interaction matrices and imputes missing or weak signals. The enhanced matrices improve the recovery of topologically associating domains and chromatin loops in bulk Hi-C data and strengthen cell-clustering performance in single-cell Hi-C applications. This study illustrates how transformer architectures can be adapted from sequential data analysis to structured genomic matrices while preserving biologically meaningful chromosomal features.

Transformer models were also applied to regulatory genomics by Kravchuk et al. [4], who investigated sequence-only prediction of super-enhancers in human cell lines. Their framework uses a genomic language model to process extended DNA sequences and distinguish super-enhancers from conventional enhancers. The analysis further examines attention patterns to identify sequence features associated with regulatory classification and their relationships with epigenetic landscapes. The ability to predict regulatory elements from sequence alone is especially valuable when comprehensive cell-type-specific epigenomic measurements are unavailable. More broadly, this work demonstrates the potential of genomic language models to learn regulatory information directly from nucleotide sequences.

## 3. Biology-Informed Learning and Optimization

The integration of biological knowledge into AI models represents another major theme of this Special Issue. Purely data-driven approaches may identify statistical associations without capturing the biological relationships that generate them. Biology-informed models can constrain the solution space, improve interpretability and increase the likelihood that computational predictions are biologically plausible.

Wang et al. proposed a biology-informed non-negative matrix factorization framework for drug repositioning [5]. Their method integrates drug- and disease-similarity networks through graph-regularized optimization, allowing latent drug–disease associations to reflect known biological relationships. Benchmarking and case studies supported the predictive performance and biological plausibility of the inferred associations. The study illustrates how established mathematical techniques, such as matrix factorization, can be strengthened through the explicit incorporation of biological context. Such approaches are particularly important in drug repositioning, where predictions must be evaluated not only statistically but also in relation to molecular pathways, disease mechanisms, and existing pharmacological evidence.

Chen and Li introduced K-volume clustering for high-dimensional and structured biological data, including single-cell RNA-sequencing datasets [6]. Instead of defining clusters solely through distances to centroids or pairwise similarities, the method uses convex volume as a geometrically interpretable optimization criterion. It jointly considers hierarchical organization and the number of clusters through nonlinear optimization. This contribution reflects an important direction in biological AI: developing objective functions that represent the structural characteristics of biological data rather than directly transferring generic clustering assumptions from other domains.

Together, these studies demonstrate that AI for complex biological systems need not be limited to increasingly deep or parameter-rich neural architectures. Mathematical modeling, geometric optimization, network regularization and biologically informed objective functions remain essential components of modern computational biology. In many applications, hybrid approaches that combine domain knowledge with machine learning may provide better interpretability, robustness and translational relevance than unconstrained black-box prediction.

## 4. AI for Biological Classification and Translational Discovery

AI applications in this collection extend beyond omics data, expanding into biological imaging, taxonomy, infectious disease, and translational research. Kumru et al. developed a hybrid deep learning framework for classifying morphologically similar puffball species using convolutional and transformer-based image architectures [7]. Accurate fungal identification can be challenging because distinct species may share highly similar macroscopic characteristics. By comparing several modern image-classification architectures on a balanced and augmented dataset, the study demonstrates the value of deep learning for automated taxonomic identification. Such tools could support biodiversity research, ecological monitoring, citizen science, and conservation, although their deployment requires careful evaluation of data independence, environmental variability, and generalization to images collected outside the original training distribution.

Izhari et al. applied immunoinformatics, structural modeling, molecular docking and molecular dynamics simulations to the design and computational evaluation of a multiepitope vaccine candidate targeting the extracellular enveloped form of the monkeypox virus [8]. Their workflow integrates antigen annotation, epitope selection, physicochemical evaluation, immune–receptor docking, structural validation, population coverage analysis and simulated immune responses. This work illustrates how computational pipelines can prioritize candidate vaccine constructs before laboratory experimentation. At the same time, it emphasizes that computational predictions represent an early stage of translational development and must ultimately be validated through in vitro, in vivo, and clinical studies.

These studies reflect the expanding scope of AI-enabled biological research. Images, molecular structures, immune epitopes and ecological observations can all be represented computationally, but each modality requires domain-specific modeling assumptions and validation strategies. The success of AI in these areas depends not only on algorithmic performance but also on representative data collection, biological plausibility and prospective experimental confirmation.

## 5. Methodological Rigor, Reproducibility, and Responsible Evaluation

The comment–reply exchange included in this Special Issue highlights the importance of rigorous experimental design in biological AI [9,10]. The discussion concerns statistical independence between training and testing data, the risk of selection bias during preprocessing and hyperparameter optimization, shortcut learning, and the calibration of predicted probabilities. These considerations are broadly applicable to biological machine learning, particularly when datasets contain multiple observations from the same specimen, collector, experimental batch, site, patient, or biological replicate.

Randomly splitting individual observations may create data leakage when related samples appear in both training and test sets. Consequently, data partitioning should often be performed at the level of the independent biological or experimental unit. Similarly, feature selection, data normalization, augmentation and hyperparameter optimization should be conducted within properly nested training procedures rather than using information from the final test set. External validation on independent datasets remains one of the strongest approaches for evaluating generalizability.

Interpretability and calibration are also critical. A model may achieve high classification accuracy by learning background features, laboratory artifacts, imaging conditions, or other shortcuts that are correlated with labels but unrelated to the biological phenomenon of interest. Explainable AI methods can assist in detecting such behavior, although explanations themselves require careful interpretation. For applications involving medical, ecological, or experimental decisions, probability calibration and uncertainty estimation may be as important as discrimination accuracy.

The inclusion of this methodological exchange strengthens the Special Issue by emphasizing that progress in biological AI requires transparent evaluation and constructive scientific discussion. Rigorous benchmarking should include strong baselines, appropriate data partitions, sensitivity analyses, uncertainty estimates, reproducible code and parameters, and validation using independent biological evidence.

## 6. Emerging Directions and Future Plans

The articles in this Special Issue collectively suggest several directions for the continued development of AI for complex biological systems. First, biological interpretability should be treated as a primary objective rather than an optional post hoc analysis. Mechanistically interpretable models, attention analysis, network-derived parameters, and biology-informed regularization can help connect predictions to biological hypotheses. Second, multimodal integration will become increasingly important. Biological mechanisms cannot usually be understood through a single data modality. Future models will need to integrate genomic sequences, gene expression, chromatin accessibility, three-dimensional genome organization, proteomics, imaging, clinical measurements, and environmental information while explicitly representing uncertainty and missing data. Third, the field must balance model scale with efficiency and scientific value. Large foundation models and generative AI systems offer substantial opportunities, but compact models may outperform or match them in specialized, low-data settings while requiring fewer computational resources. Model selection should therefore consider accuracy, interpretability, energy consumption, training data requirements, and accessibility in addition to architectural novelty. Fourth, AI models should increasingly move from retrospective pattern recognition toward causal and dynamical modeling. Perturbation response data, longitudinal measurements, neural differential equations, agent-based models and hybrid mechanistic–AI frameworks can support the prediction of how biological systems respond to genetic, pharmacological, or environmental interventions. Finally, computational predictions must be connected to experimental and translational validation. AI can prioritize hypotheses, identify candidate mechanisms and reduce large search spaces, but biological conclusions require independent data and, when possible, experimental confirmation. Close collaboration among computational scientists, biologists, clinicians and domain experts will remain essential. Building on these insights, we will continue to create Special Issues that advance interdisciplinary research and promote innovative applications of artificial intelligence and deep learning in biology and biomedicine.

## 7. Conclusions

The contributions to the Special Issue, titled “Artificial Intelligence Research for Complex Biological Systems”, demonstrate the breadth and rapid evolution of AI-enabled biological research. The studies range from interpretable single-cell perturbation modeling and generative enhancement of chromatin interaction data to regulatory sequence prediction, geometry-based clustering, biology-informed drug repositioning, fungal image classification, and computational vaccine design. The accompanying review and methodological discussion place these advances within a broader context and emphasize the need for interpretability, reproducibility, rigorous validation, and responsible model evaluation. AI is becoming an increasingly powerful component of biological discovery, but its greatest impact will arise when computational innovation is closely integrated with biological knowledge. Models that are accurate, interpretable, efficient, reproducible, and experimentally testable will be best positioned to advance our understanding of complex biological systems and translate computational discoveries into meaningful biomedical and ecological applications.

As Guest Editors, we would like to thank all authors for contributing their research and perspectives to this Special Issue. We also sincerely thank the reviewers for their careful evaluations and constructive recommendations and the editorial staff of *Biology* for their professional support throughout the publication process. We hope that this collection will stimulate further interdisciplinary research and encourage the development of innovative, rigorous, and biologically informed AI approaches.
